# isoCNV: in silico optimization of copy number variant detection from targeted or exome sequencing data

**DOI:** 10.1186/s12859-021-04452-6

**Published:** 2021-10-29

**Authors:** Rosa Barcelona-Cabeza, Walter Sanseverino, Riccardo Aiese Cigliano

**Affiliations:** 1 Sequentia Biotech, Carrer de Valencia, Barcelona, Spain; 2 Departamento de Matemáticas, Escuela Técnica Superior de Ingeniería Industrial de Barcelona (ETSEIB), Universitat Politècnica de Catalunya (UPC), Diagonal 647, Barcelona, Spain

**Keywords:** Copy number variants, CNV, Optimization, NGS, WES, TS

## Abstract

**Background:**

Accurate copy number variant (CNV) detection is especially challenging for both targeted sequencing (TS) and whole‐exome sequencing (WES) data. To maximize the performance, the parameters of the CNV calling algorithms should be optimized for each specific dataset. This requires obtaining validated CNV information using either multiplex ligation-dependent probe amplification (MLPA) or array comparative genomic hybridization (aCGH). They are gold standard but time-consuming and costly approaches.

**Results:**

We present isoCNV which optimizes the parameters of DECoN algorithm using only NGS data. The parameter optimization process is performed using an in silico CNV validated dataset obtained from the overlapping calls of three algorithms: CNVkit, panelcn.MOPS and DECoN. We evaluated the performance of our tool and showed that increases the sensitivity in both TS and WES real datasets.

**Conclusions:**

isoCNV provides an easy-to-use pipeline to optimize DECoN that allows the detection of analysis-ready CNV from a set of DNA alignments obtained under the same conditions. It increases the sensitivity of DECoN without the need for orthogonal methods. isoCNV is available at https://gitlab.com/sequentiateampublic/isocnv.

## Background

Next generation sequencing (NGS) technologies have become increasingly a standard application for large-scale DNA sequencing because of their high throughput and cost-effectiveness. Both targeted sequencing (TS) and whole‐exome sequencing (WES) are used as effective assays to detect single-nucleotide variations (SNVs) and small insertion and deletion (indels) [1–5]. Copy-Number Variants (CNV) have been associated with a wide collection of diseases including Parkinson [6, 7], Autism [8, 9], or Alzheimer [10] and some were proven to be the genetic cause of several hereditary diseases [11]. However, accurate detection of copy-number variants (CNV) from NGS data is still challenging due to several technical issues including short read length and GC-content bias [12]. Furthermore, compared to whole-genome sequencing (WGS), TS and WES data introduce more biases due to hybridization and to a non-uniform read-depth distribution among regions [13–15] that make CNV detection even more difficult. Nevertheless, TS and WES can offer greater depth at a lower price and a faster and less complex data analysis.

Many tools have been developed for CNV detection using TS or WES data [13, 16–20]. Among them there is DECoN [18], which has shown a high performance with NGS panel data [21, 22]. DECoN is based on read depth data to call CNV and is the result of modification and optimization of ExomeDepth v1.0.0 [16]. However, its performance is highly dependent on the selected parameters which should be tuned for each specific dataset to maximize sensitivity [22] and should not be used directly with data produced differently, i.e. with different sequencing technologies, targeting probes or capture protocol [22].

Parameter optimization can be performed using an optimizer from the CNVbenchmarkeR framework [22]. However, the parameter optimization process requires a CNV validation set, which is usually generated using either multiplex ligation-dependent probe amplification (MLPA) or array comparative genomic hybridization (aCGH). They are the gold standard methods [23], but both are time-consuming and costly approaches.

Here we present the isoCNV pipeline, which performs *in silico* optimization of DECoN parameters to maximize its sensitivity using only NGS data. We propose to obtain the CNV validation set from the overlapping calls of three CNV calling tools: CNVkit [24], panelcn.MOPS [19] and DECoN. We show that our tool increases the sensitivity of DECoN for both TS and WES data. In addition, it is easy to implement and allows to obtain analysis-ready CNV from DNA sequencing read alignments in BAM format [25].

## Implementation

**Fig. 1:**
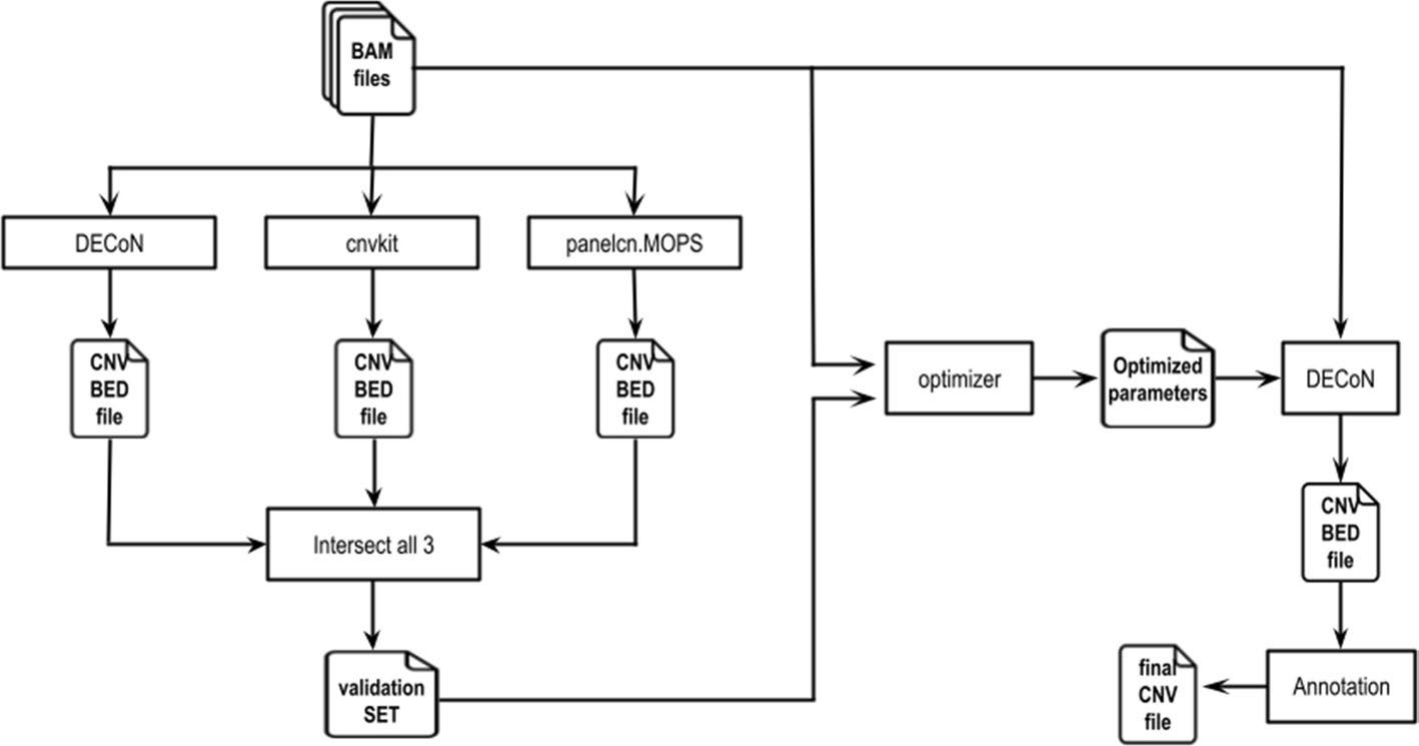
Overview of the pipeline. (1) CNV calling is performed using three different tools: CNVkit, panelcn.MOPS and DECoN with default parameters. (2) The CNV validation set is obtained from the overlapping calls of the three CNV calling tools. (3) DECoN algorithm is executed using up to 22 different values for each parameter. The results obtained with each combination of parameters are compared with the validated set to obtain optimized parameters. (4) CNV calling is performed using DECoN with optimized parameters. (5) CNV are annotated

Our pipeline is a Python 3.7 software package comprising a command-line program, isoCNV.py. The input to the program is a batch of BAM files from TS or WES samples obtained under the same conditions and the regions of interest (ROI) in BED format that should correspond with the capture bait locations. The program is completely modular and allows to run the complete pipeline in batch or perform the step-by-step analysis. The pipeline consists of 5 main steps: individual CNV calling using three different algorithms, creation of an *in silico* validation dataset, parameter optimization, CNV calling with optimized parameters and CNV annotation (Fig. 1).

### Datasets

A targeted and a whole-exome sequencing dataset were selected to evaluate the performance of isoCNV: ICR96 exon CNV validation series [26] and NimbleGen set [27], respectively. ICR96 exon CNV validation series includes 96 samples and NimbleGen set includes 34 samples. Both datasets have available validated CNV information, ICR96 have been validated by MLPA and NimbleGen by SNP microarray [28]. ICR96 exon CNV validation series can be downloaded from European-Genome phenome Archive (EGA), which is hosted by the EBI and the CRG, under accession number EGAS00001002428. The FASTQ files for NimbleGen dataset can be accessed through the Sequence Read Archive (SRA) [29] under accession number SRP010920.

### Data preprocessing

All samples were aligned to the GRCh37 human genome assembly using BWA-MEM algorithm developed by Wellcome Trust Sanger Institute [30]. Sentieon sort utility [31] was used to sort and index BAM files. Then, duplicate reads were removed and base quality score recalibration (BQSR) was performed using the Sentieon utilities [31]. Sentieon is a commercial variant caller that is designed as an accelerated software for Genome Analysis Toolkit (GATK) [32].

### Individual CNV calling

Preliminary identification of copy number variants is performed using three different CNV callers: DECoN v1.0.2 [22] with default parameters, CNVkit v0.9.6 [24] and panelcn.MOPS v1.12.0 [19]. Since the CNV identification method is based on depth of coverage, the gender of the samples is a critical factor to determine variations in copy number of sex chromosomes. Therefore, one of the mandatory inputs to perform the analysis is the gender of the samples, which can be provided by the user or it will be automatically inferred using the CNVkit gender tool.

Default parameters are applied to perform the CNV calling using DECoN but with the modifications described below. DECoN creates a reference set for each sample of interest consisting only of those samples which are well correlated [22]. Hence related individuals should be excluded from the reference, otherwise common CNV in the family would not be detected. For this reason, a list of related samples can be provided in order to automatically exclude them from the reference set of their relatives in order not to lose CNV of the family. In addition, it has been found that the optimum size of the reference set is between 5 and 10 samples [16] so DECoN has been modified to only accept a maximum of 10 samples as reference. Moreover, it should be noted that by default, CNV calling is performed separately between male and female samples, thus allowing the detection of CNV in the sex chromosomes. However, if there are less than 5 female or male samples, all samples are analyzed in a single batch, disabling a reliable CNV calling on sex chromosomes. Optionally, only sex chromosomes can be analyzed separately between male and female samples, using the “batch2” option of isoCNV. Regions of interest (ROI) will be dropped if they are below the default minimum median coverage threshold (100) for any sample (measured across all ROI in the target) or region (measured across all samples). CNV will be filtered out from samples that do not meet either the minimum coverage threshold (100) or the minimum correlation threshold (0.98). Samples which do not have a high correlation with other samples in the set are likely to have suboptimal detection across the entire target. These two types of filters have been added as options so that the user can easily select whether to apply them.

Regarding CNV calling with CNVkit, default parameters are also applied except for filtering where the ‘cn’ method is applied instead of ‘ci’. Here a single reference set is created for all samples, it will be composed of all female samples in the batch with a standard deviation (SD) between − 2 and 2. Such a reference set will be only modified if the sample of interest is female, in which case it will be excluded from the reference. Two exceptions should be noted in the creation of the reference set: (1) if there are less than 5 female samples, then males will be the ones used as reference and (2) in the case that there are less than 5 females and less than 5 males, then all samples will be used as a reference and CNV in Y chromosome will be unreliable. Furthermore, the thresholds used by CNVkit to define copy numbers 0 and 1 were modified to be more restrictive: for CN0 the threshold range (log_2_ value up to) has changed from log_2_ ≦ − 1.1 to log_2_ ≦ − 2 and for CN1 from − 1.1 < log_2_ ≦ − 0.4 to − 2 < log_2_ ≦ − 0.4. The precise copy number values obtained by CNVkit (0. 1, 2, 3, etc) are then converted to deletion (DEL) or duplication (DUP) taking into account the gender of the sample of interest and the gender of the references.

The identification of CNV with panelcn.MOPS is also carried out using the default parameters of the tool. As with DECoN, the analysis is carried out in two groups, one with the female samples and another with the male ones, unless there are less than 5 females or males that all samples will be analyzed together. ROI are excluded from the analysis if marked as “low quality” by panelcn.MOPS: their median read count across all samples does not exceed the minimum default threshold (30) or if their read count shows a high variation across all samples as marked by the default behaviour of the algorithm.

### In silico validation dataset

The in silico validation dataset is composed of the overlapping calls of the three CNV calling tools (DECoN with default parameters, CNVkit and panelcn.MOPS). In order to compare the results obtained by the three calling tools and create an in silico validation dataset, the output of each tool is normalized to a single format, a tab-delimited BED file. This file contains five columns corresponding to chromosome, start position of the CNV, end position of the CNV, CNV type (DEL or DUP) and samplename. Using BEDTools utilities v2.29.2 [33] and pybedtools Python library v0.8.1 [34], the overlapping CNV between call sets from the three algorithms are selected if meet two criteria (1) at least 60% of overlap with one of the call sets from the algorithms and (2) a minimum size equivalent to the mean size of the target ROIs. If one of the tools reports no CNV in any sample, only the output of the other two algorithms is used to create the in silico validation set.

### Parameter optimization

Parameter optimization is performed using the feature optimizer from CNVbenchmarkeR framework [22]. From a validated dataset, it executes DECoN algorithm against the dataset using up to 22 different values for each parameter. The results obtained with each combination of parameters are compared with the validated copy number states in order to obtain optimized parameters for the dataset.

Here, the validated CNV are the ones obtained in silico from the overlapped calls between DECoN, CNVkit and panelcn.MOPS (the in silico validation dataset). Nevertheless, it is also necessary to provide validated information about regions with a normal copy number state. To do this, all regions where a CNV has been found (and has been validated in silico) in any of the samples from the dataset are selected as validated regions, and then, a normal copy number state is assigned to each validated region with no validated CNV.

The DECoN parameters subject to optimization are the following: (1) the minimum correlation threshold between a test sample and any other sample to be considered well correlated, (2) the minimum median coverage for any sample or ROI to be considered well-covered and (3) the transition probability between normal copy number state and either deletion or duplication state in the hidden Markov model.

The identification of copy number variants is performed using DECoN algorithm using the same approach applied to create the CNV validation dataset: (1) a maximum of 10 samples are used as reference per sample, (2) related individuals are excluded from the reference set and (3) female and male samples are processed separately. Nevertheless, instead of using the default parameters, the optimized ones obtained in the previous step are used to perform the analysis.

The results are the final copy number variants, which are normalized in BED format with the following columns: chromosome, start position of the CNV, end position of the CNV, CNV type (DEL or DUP), sample name, reads ratio and the precise copy number value. Reads ratio corresponds to the one calculated by DECoN algorithm and copy number values are calculated based on the reads ratio (Table 1).

**Table 1:** The reads ratio thresholds map to integer copy numbers

Threshold range	Copy number value
$ReadsRatio\leqq 0.1$	0
$0.1<ReadsRatio\leqq 0.8$	1
$0.8<ReadsRatio\leqq 1.2$	2
$1.2<ReadsRatio\leqq 1.8$	3
$1.8<ReadsRatio\leqq 2.2$	4
2.2 < *ReadsRatio*	*ReadsRatio* * 2

### CNV annotation

Finally, CNV are annotated using the AnnotSV tool [35]. AnnotSV provides numerous relevant annotations: genes-based annotation (OMIM, Haploinsufficiency, Gene intolerance, etc), annotation with features overlapping the CNV (databases of known CNV such as gnomAD or 1000 genomes), annotation with features overlapped with the CNV (pathogenic SV from dbVar, promoters, etc) and annotation of the breakpoints (GC content, segmental duplications, etc). Therefore, it classifies CNVs according to their pathogenicity into one of the 5 classes proposed by the American College of Medical Genetics and Genomics (ACMG) guidelines: benign, likely benign, variant of unknown significance (VUS), likely pathogenic or pathogenic. All of this makes it easier for prioritization of copy number variants of interest.

### Benchmark evaluation metrics

The performance of isoCNV was evaluated per region of interest (ROIs). Such ROIs correspond to the target bed file of each dataset and were treated as independent entities. If the tool matched the result of the validation information was classified as true positive (TP) or true negative (TN). If the tool identified a CNV not present in the validation information was a false positive (FP) and if the tool missed a validated CNV was a false negative (FN).

Furthermore, the performance of isoCNV was evaluated taking the no calls into account. This is due to the fact that in a real diagnostic scenario, all regions where there is no call should be confirmed by an orthogonal method.

## Results

### In silico validation dataset

**Fig. 2:**
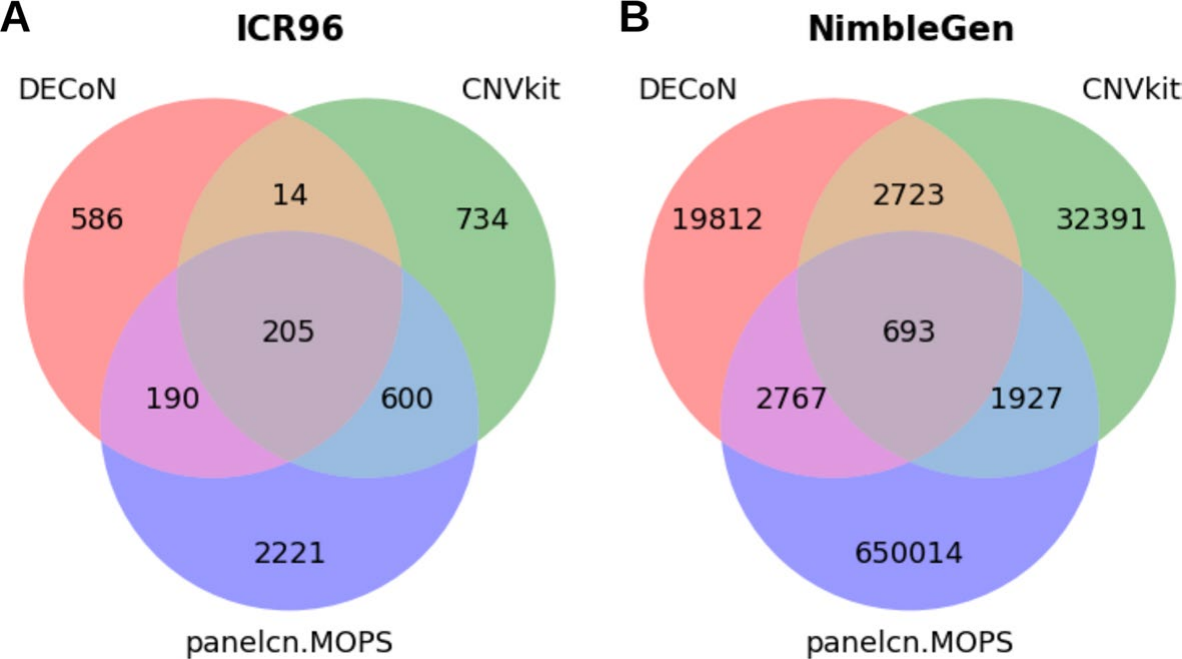
Counts of the CNV per ROI detected by three callers. **A** Venn Diagram of the CNV in ICR96 dataset. **B** Venn Diagram of the CNV in NimbleGen dataset

The total copy number variants identified per ROI, for each calling tool and dataset, is shown in a Venn diagram (Fig. 2). It is shown that the total number of CNVs per ROI varies across algorithms. In both datasets, panelcn.MOPS identified the greatest number of CNVs whereas DECoN identified the least number. The overlapped CNVs per ROI between the three call sets were 205 in the TS dataset (ICR96) and 693 in the WES dataset (NimbleGen) (Fig. 2). From these, the validation dataset was composed from the ones that overlapped at least 60% with one of the call sets from the algorithms and that had a minimum size equivalent to the mean size of the target ROIs. Hence, 72 validated CNVs were obtained in ICR96 and 388 in NimbleGen.

After the regions with normal copy number state were attached to the validation set, such validation set could be compared to the real copy number information obtained by MLPA in ICR96 and by SNP microarray in NimbleGen set (Table 2). For both datasets, specificity was 1 as no FP were identified, while sensitivity was quite low as a high number of FN were found. These results were expected, due to the stringent filters that we apply to define a copy number as validated before proceeding to the optimization step.

**Table 2:** Benchmark results for the individual callings and the in silico validation dataset

Dataset	Method	TP	TN	FP	FN	Total	Sensitivity	Specificity	PPV	NPV	F-score
ICR96	DECoN	247	27,330	60	49	27,686	0.8345	0.9978	0.8046	0.9982	0.8192
	CNVkit	205	27,225	91	165	27,686	0.5541	0.9967	0.6827	0.9940	0.6156
	panelcn.MOPS	278	27,061	18	329	27,686	0.4580	0.9993	0.9392	0.9880	0.6157
	In silico validation	58	27,390	0	238	27,686	0.1959	1	1	0.9914	0.3277
NimbleGen	DECoN	220	7236	43	1138	8637	0.1620	0.9941	0.8365	0.8641	0.2714
	CNVkit	777	7274	582	4	8637	0.9949	0.9259	0.5717	0.9995	0.7262
	panelcn.MOPS	736	6891	619	391	8637	0.6531	0.9176	0.5432	0.9463	0.5931
	In silico validation	30	7278	0	1329	8637	0.0220	1	1	0.8456	0.0432

### Benchmark evaluation

**Fig. 3:**
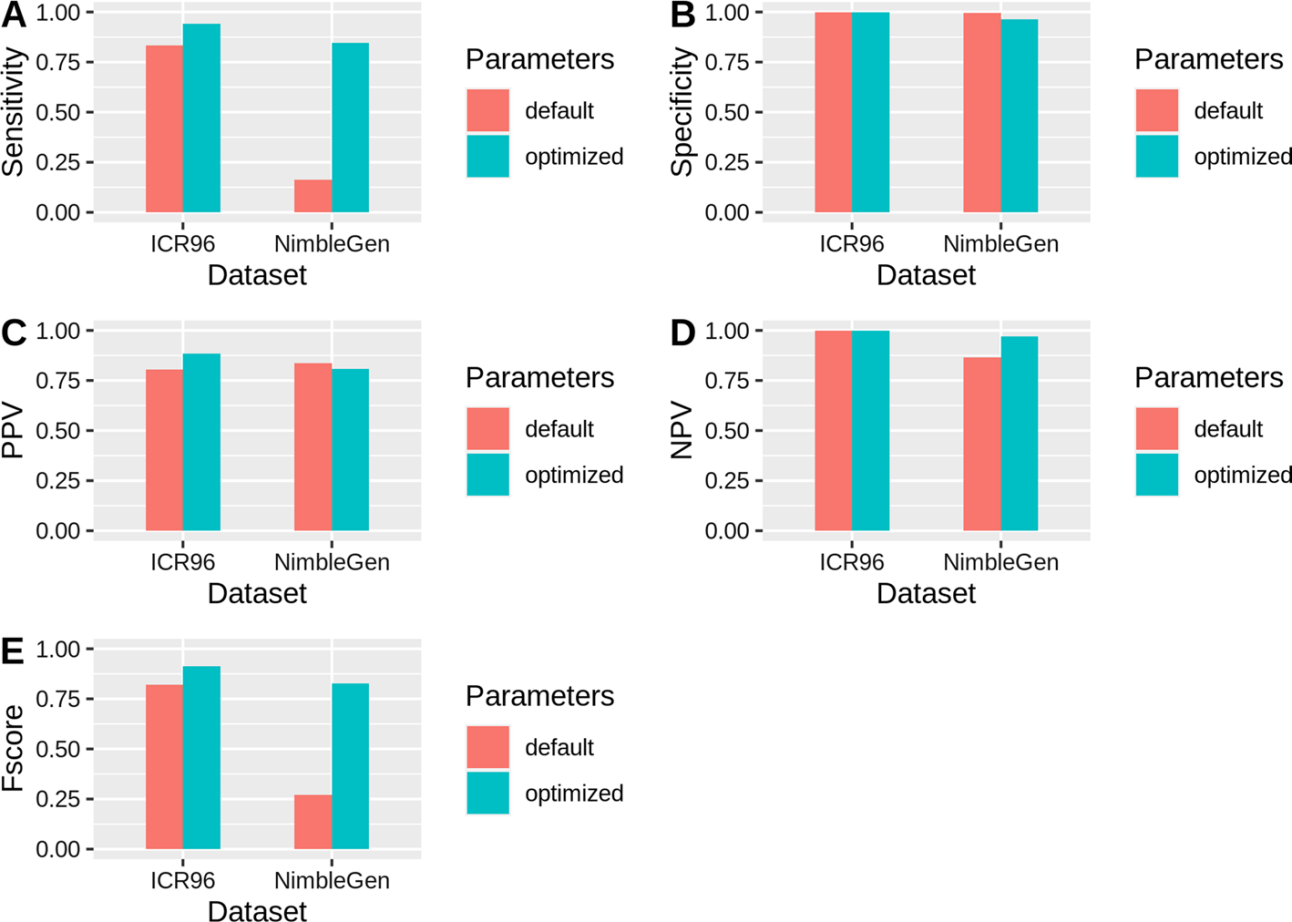
Benchmark results with default and optimized parameters. Shows sensitivity, specificity, PPV, NPV and F-score when executing DECoN with the optimized parameters in comparison to the default parameters

After the parameter optimization of DECoN, 597 CNV were identified in ICR96 and 125601 in NimbleGen. There was an increase in sensitivity and F-score for both dataset but especially for NimbleGen set where there was a major improvement in sensitivity (from 16.2 to 84.5%) and F-score (from 27.1 to 82.7%) by slightly decreasing specificity (from 99.4 to 96.3%) (Fig. 3, Table 3). Negative Predictive Value (NPV) was higher than the Positive Predictive Value (PPV) before and after optimization process in both datasets (Fig. 3, Table 3) as expected in unbalanced datasets with a much larger number of negative elements (no calls) than positive ones.

**Table 3:** Benchmark results with default and optimized parameters

Dataset	Parameters	TP	TN	FP	FN	Total	Sensitivity	Specificity	PPV	NPV	F-score
ICR96	Default	247	27,330	60	49	27,686	0.8345	0.9978	0.8046	0.9982	0.8192
	Optimized	279	27,354	36	17	27,686	0.9426	0.9987	0.8857	0.9994	0.9133
NimbleGen	Default	220	7236	43	1138	8637	0.1620	0.9941	0.8365	0.8641	0.2714
	Optimized	1147	7009	271	210	8637	0.8452	0.9628	0.8089	0.9709	0.8267

To evaluate if parameter optimization of DECoN allows to identify new CNVs only predicted by the other two methods (CNVkit and panelcn.MOPS) when default parameters are used, the unique CNVs of CNVkit (identified by CNVkit but not by DECoN with default parameters) have been obtained and compared to the final CNVs (identified by DECoN with optimized parameters) and found 86 and 2727 CNVs in common in the ICR96 and NimbleGen dataset, respectively. The same approach has been applied to the unique CNVs of panelcn.MOPS and 88 (ICR96) and 68569 (NimbleGen) CNVs have been found in the final CNVs that were not identified initially by DECoN with default parameters.

**Fig. 4:**
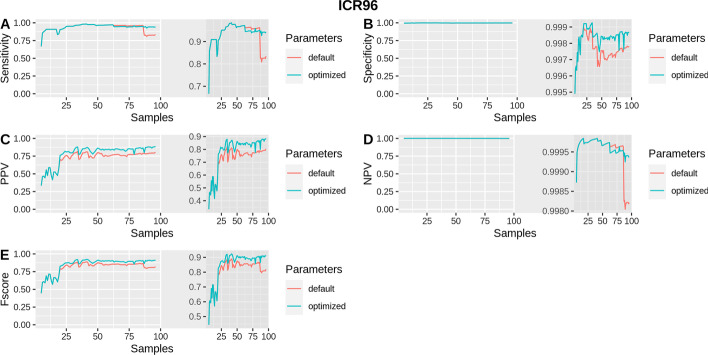
Benchmark results with default and optimized parameters when analyzing different numbers of samples in ICR96. Shows sensitivity, specificity, PPV, NPV and F-score when executing DECoN for different numbers of samples (from 5 to 96) with the optimized parameters in comparison to the default parameters

**Fig. 5:**
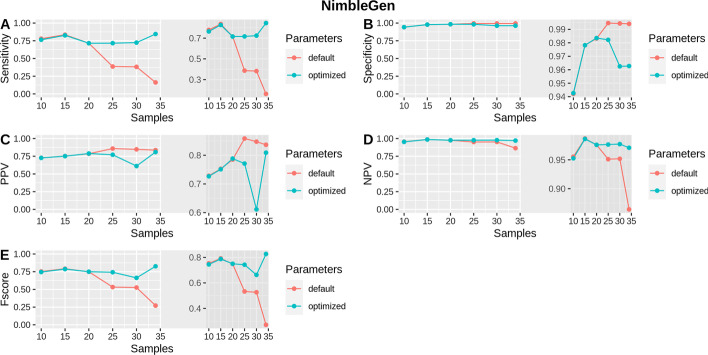
Benchmark results with default and optimized parameters when analyzing different numbers of samples in NimbleGen. Shows sensitivity, specificity, PPV, NPV and F-score when executing DECoN with the optimized parameters in comparison to the default parameters for different numbers of samples adding 5 each time

In addition, the performance of isoCNV was evaluated depending on the number of samples analyzed. This relates to the reference set as samples with a better correlation or a higher coverage may be included and could improve the performance of DECoN. The ICR96 set reached almost 100% specificity and NPV independently of the number of samples with both default and optimized parameters (Fig. 4). An improvement in PPV and F-score can be observed in the ICR96 set when at least 20 samples were analyzed together and then, from 24 samples, both PPV and F-score remained fairly constant, being always higher when executing DECoN with optimized parameters (Fig. 4). The sensitivity in the ICR96 set also remained quite constant and above 80% when at least 6 samples were analyzed with optimized parameters, whereas there was a decrease in the sensitivity when more than 86 samples were analyzed with default parameters (Fig. 4). The NimbleGen set showed a fairly constant sensitivity, specificity, PPV, NPV and F-score with optimized parameters (Fig. 5). However, sensitivity, F-score and NPV decreased considerably when analyzing more than 20 samples using default parameters (Fig. 5).

## Conclusions

We presented isoCNV, an automated pipeline to optimize DECoN algorithm using only NGS data. It allows the detection of analysis-ready CNV from a set of DNA alignments and their corresponding capture bait locations. It has been shown to improve sensitivity of DECoN in both TS and WES data, which is especially critical when this tool is used as a screening step in a diagnostic strategy. We thus hope to reduce the number of assays required per patient to reach a diagnosis as orthogonal methods, such as MLPA or aCGH, are not required.

## Availability and requirements

- Project name: isoCNV.
- Project home page: https://gitlab.com/sequentiateampublic/isocnv.
- Operating system: Platform independent.
- Programming language: Python3.
- Other requirements: Python packages (pandas, biopython, pybedtools), BEDtools, DECoN, CNVkit, panelcn.MOPS, CNVbenchmarkeR, AnnotSV.
- License: CC NC.
- Any restrictions to use by non-academics: license needed.

## Data Availability

Source code is available at https://gitlab.com/sequentiateampublic/isocnv. ICR96 exon CNV validation series can be downloaded from European-Genome phenome Archive (EGA) under accession number EGAS00001002428 at https://ega-archive.org/studies/EGAS00001002428. NimbleGen dataset can be downloaded from Sequence Read Archive (SRA) under accession number SRP010920 at https://www.ncbi.nlm.nih.gov/sra/SRP010920.

## Abbreviations

aCGH: Array Comparative Genomic Hybridization
ACMG: American College of Medical Genetics and Genomics
BQSR: Base Quality Score Recalibration
CNV: Copy number variants
FN: False negative
FP: False positive
GATK: Genome Analysis Toolkit
indels: Small insertion and deletion
MLPA: Multiplex Ligation-dependent Probe Amplification
NGS: Next generation sequencing
NPV: Negative predictive value
PPV: Positive predictive value
ROI: Region of interest
SD: Standard deviation
SNV: Single-nucleotide variations
TN: True negative
TP: True positive
TS: Targeted sequencing
VUS: Variant of unknown significance
WES: Whole exome sequencing
WGS: Whole genome sequencing

## References

[1] Huang L, Yang J, Xu S, Mao Y, Lee DY, Yang J (2018). Whole exome sequencing identifies mutations of multiple genes in a Chinese cohort of 95 sporadic probands with presumptive retinitis pigmentosa. J Bio-X Res.

[2] Tsaousis GN, Papadopoulou E, Apessos A, Agiannitopoulos K, Pepe G, Kampouri S (2019). Analysis of hereditary cancer syndromes by using a panel of genes: novel and multiple pathogenic mutations. BMC Cancer.

[3] Herodež ŠS, Stangler Herodež Š, Marčun Varda N, Kokalj Vokač N, Krgović D (2020). De novo KMT2D heterozygous frameshift deletion in a newborn with a congenital heart anomaly. Balk J Med Genet.

[4] Okano T, Imai K, Naruto T, Okada S, Yamashita M, Yeh T-W (2020). Whole-exome sequencing-based approach for germline mutations in patients with inborn errors of immunity. J Clin Immunol.

[5] Cortese A, Wilcox JE, Polke JM, Poh R, Skorupinska M, Rossor AM (2020). Targeted next-generation sequencing panels in the diagnosis of Charcot-Marie-Tooth disease. Neurology.

[6] Pankratz N, Dumitriu A, Hetrick KN, Sun M, Latourelle JC, Wilk JB (2011). Copy number variation in familial Parkinson disease. PLoS ONE.

[7] La Cognata V, Morello G, D’Agata V, Cavallaro S (2017). Copy number variability in Parkinson’s disease: assembling the puzzle through a systems biology approach. Hum Genet.

[8] Vicari S, Napoli E, Cordeddu V, Menghini D, Alesi V, Loddo S (2019). Copy number variants in autism spectrum disorders. Prog Neuropsychopharmacol Biol Psychiatry.

[9] Velinov M (2019). Genomic copy number variations in the autism clinic-work in progress. Front Cell Neurosci.

[10] Brouwers N, Van Cauwenberghe C, Engelborghs S, Lambert J-C, Bettens K, Le Bastard N (2012). Alzheimer risk associated with a copy number variation in the complement receptor 1 increasing C3b/C4b binding sites. Mol Psychiatry.

[11] Zhang F, Gu W, Hurles ME, Lupski JR (2009). Copy number variation in human health, disease, and evolution. Annu Rev Genomics Hum Genet.

[12] Teo SM, Pawitan Y, Ku CS, Chia KS, Salim A (2012). Statistical challenges associated with detecting copy number variations with next-generation sequencing. Bioinformatics.

[13] Krumm N, Sudmant PH, Ko A, O’Roak BJ, Malig M, Coe BP (2012). Copy number variation detection and genotyping from exome sequence data. Genome Res.

[14] Kadalayil L, Rafiq S, Rose-Zerilli MJJ, Pengelly RJ, Parker H, Oscier D (2015). Exome sequence read depth methods for identifying copy number changes. Brief Bioinform.

[15] Kebschull JM, Zador AM (2015). Sources of PCR-induced distortions in high-throughput sequencing data sets. Nucleic Acids Res.

[16] Plagnol V, Curtis J, Epstein M, Mok KY, Stebbings E, Grigoriadou S (2012). A robust model for read count data in exome sequencing experiments and implications for copy number variant calling. Bioinformatics.

[17] Samarakoon PS, Sorte HS, Kristiansen BE, Skodje T, Sheng Y, Tjønnfjord GE (2014). Identification of copy number variants from exome sequence data. BMC Genomics.

[18] Fowler A, Mahamdallie S, Ruark E, Seal S, Ramsay E, Clarke M (2016). Accurate clinical detection of exon copy number variants in a targeted NGS panel using DECoN. Wellcome Open Res.

[19] Povysil G, Tzika A, Vogt J, Haunschmid V, Messiaen L, Zschocke J (2017). panelcn.MOPS: copy-number detection in targeted NGS panel data for clinical diagnostics. Hum Mutat.

[20] Jiang Y, Wang R, Urrutia E, Anastopoulos IN, Nathanson KL, Zhang NR (2018). CODEX2: full-spectrum copy number variation detection by high-throughput DNA sequencing. Genome Biol.

[21] Roca I, González-Castro L, Fernández H, Couce ML, Fernández-Marmiesse A (2019). Free-access copy-number variant detection tools for targeted next-generation sequencing data. Mutat Res.

[22] Moreno-Cabrera JM, Del Valle J, Castellanos E, Feliubadaló L, Pineda M, Brunet J (2020). Evaluation of CNV detection tools for NGS panel data in genetic diagnostics. Eur J Hum Genet.

[23] Kerkhof J, Schenkel LC, Reilly J, McRobbie S, Aref-Eshghi E, Stuart A (2017). Clinical validation of copy number variant detection from targeted next-generation sequencing panels. J Mol Diagn.

[24] Talevich E, Shain AH, Botton T, Bastian BC (2016). CNVkit: genome-wide copy number detection and visualization from targeted DNA sequencing. PLoS Comput Biol.

[25] Li H, Handsaker B, Wysoker A, Fennell T, Ruan J, Homer N (2009). The sequence alignment/map format and SAMtools. Bioinformatics.

[26] Mahamdallie S, Ruark E, Yost S, Ramsay E, Uddin I, Wylie H (2017). The ICR96 exon CNV validation series: a resource for orthogonal assessment of exon CNV calling in NGS data. Wellcome Open Res.

[27] Sanders SJ, Murtha MT, Gupta AR, Murdoch JD, Raubeson MJ, Willsey AJ (2012). De novo mutations revealed by whole-exome sequencing are strongly associated with autism. Nature.

[28] Krumm N, Turner TN, Baker C, Vives L, Mohajeri K, Witherspoon K (2015). Excess of rare, inherited truncating mutations in autism. Nat Genet.

[29] Leinonen R, Sugawara H, Shumway M (2011). International nucleotide sequence database collaboration. The sequence read archive. Nucleic Acids Res.

[30] Li H, Durbin R (2010). Fast and accurate long-read alignment with Burrows–Wheeler transform. Bioinformatics.

[31] Freed D, Aldana R, Weber JA, Edwards JS (2017). The sentieon genomics tools—a fast and accurate solution to variant calling from next-generation sequence data. Cold Spring Harb Lab..

[32] McKenna A, Hanna M, Banks E, Sivachenko A, Cibulskis K, Kernytsky A (2010). The genome analysis toolkit: a MapReduce framework for analyzing next-generation DNA sequencing data. Genome Res.

[33] Quinlan AR, Hall IM (2010). BEDTools: a flexible suite of utilities for comparing genomic features. Bioinformatics.

[34] Dale RK, Pedersen BS, Quinlan AR (2011). Pybedtools: a flexible Python library for manipulating genomic datasets and annotations. Bioinformatics.

[35] Geoffroy V, Herenger Y, Kress A, Stoetzel C, Piton A, Dollfus H (2018). AnnotSV: an integrated tool for structural variations annotation. Bioinformatics.

